# The Benefits of Humanized Yeast Models to Study Parkinson's Disease

**DOI:** 10.1155/2013/760629

**Published:** 2013-07-01

**Authors:** V. Franssens, T. Bynens, J. Van den Brande, K. Vandermeeren, M. Verduyckt, J. Winderickx

**Affiliations:** Functional Biology, KU Leuven, Kasteelpark Arenberg 31, 3001 Heverlee, Belgium

## Abstract

Over the past decade, the baker's yeast *Saccharomyces cerevisiae* has proven to be a useful model system to investigate fundamental questions concerning the pathogenic role of human proteins in neurodegenerative diseases such as Parkinson's disease (PD). These so-called humanized yeast models for PD initially focused on **α**-synuclein, which plays a key role in the etiology of PD. Upon expression of this human protein in the baker's yeast *Saccharomyces cerevisiae*, the events leading to aggregation and the molecular mechanisms that result in cellular toxicity are faithfully reproduced. More recently, a similar model to study the presumed pathobiology of the **α**-synuclein interaction partner synphilin-1 has been established. In this review we will discuss recent advances using these humanized yeast models, pointing to new roles for cell wall integrity signaling, Ca^2+^ homeostasis, mitophagy, and the cytoskeleton.

## 1. Introduction

Parkinson's disease (PD) is the most prevalent neurodegenerative movement disorder in elderly people. The clinical features of this disease include motor deficits such as resting tremor, rigidity, bradykinesia, and postural instability. These symptoms result from the selective and progressive loss of dopaminergic neurons in the substantia nigra pars compacta and the presence of fibrillary cytoplasmic inclusions called Lewy bodies. Although early studies mainly addressed environmental factors as the cause of neuronal demise, these days the involvement of genetic risk factors is the main focus of many studies. At present 18 genetic loci (designated PARK1-18) have been associated with the development of PD, including the autosomal dominant *α*-synuclein, LRRK2, and Omi/Htra2 and the autosomal recessive Parkin, PINK1, DJ-1, and ATP13A2 [1]. *α*-Synuclein was the first gene that was found to play a role in the pathogenesis of PD. Beside the missense mutations A53T, A30P, and E46 K [2–4], duplications and triplications of this gene have been shown to result in parkinsonism [5–7]. In addition, aggregated *α*-synuclein was identified as the major component of Lewy bodies in the brains of sporadic PD patients [8]. The presynaptic protein synphilin-1, which has been identified as an *α*-synuclein interaction partner using the yeast-two-hybrid system [9], was also found to be a Lewy body constituent [10]. Since the discovery of *α*-synuclein as a key player in PD, a diverse research community has evolved, focusing on the molecular properties of this protein, and its interaction partner synphilin-1, and the cellular dysfunction that underlies *α*-synuclein-mediated pathology. A whole range of model systems have been developed to study the different levels and aspects of *α*-synuclein and synphilin-1 dysfunction. Studies in the classical animal models, like transgenic mice,* Drosophila melanogaster*, and *Caenorhabditis elegans*, have been able to model the *in vivo* aspects of the disease. However, mechanistic aspects of a disease often emerge from studies at the cellular and subcellular level. To this end, usually, mammalian cell lines are used, but recently also the budding yeast *Saccharomyces cerevisiae* has manifested itself as a valuable model to provide insight into the mechanisms of PD. Recent new findings in yeast models expressing *α*-synuclein led to new roles for cell wall integrity signaling, intracellular Ca^2+^ buildup, and mitophagy, underscoring the usefulness and power of a yeast model to uncover new aspects of PD pathology.

## 2. Humanized Yeast Models 

At first sight it might not seem very obvious to use a simple and unicellular organism such as yeast to study a complex brain disorder like PD. Indeed, the budding yeast has its limitations, as it cannot recapitulate the complex cellular interactions occurring in the human brain. Likewise, proteins and pathways required for the development of multicellular organisms, are not represented in yeast. Still, yeast cells possess strong similarities to human cells. Around 60% of the yeast genes show sequence homology to a human orthologue [11], and of the human disease-related genes, over 25% have a close homologue in yeast [12]. Importantly, yeast and human cells share fundamental aspects of eukaryotic cell biology. In many cases, yeast has even been the model system where these cellular processes and the genetic components comprising them have been elucidated. This allows a number of key processes, which are of particular interest to PD pathology, to be efficiently investigated in the well-understood yeast model. These include the mechanisms of protein folding, quality control and degradation, the components involved in the secretory pathway and vesicular trafficking, the study of mitochondrial dysfunction and oxidative stress, and even the mechanisms of cell death and survival. Finally, yeast models also possess several clear advantages compared to higher model organisms [13]. Budding yeast cultures show a rapid growth, with a doubling time of 1,5 to 3 hours. This allows a fast and easy scale-up, which is profitable for high-throughput genetic and small-molecule screens. Yeast is readily amenable to many different genetic modifications. Easy DNA transformation and the availability of a host of selectable markers allow the introduction of multiple self-replicating plasmids. Moreover, stable and highly specific introduction of genes, modifications, and markers in the genome, through introduction of DNA sequences by homologous recombination, is highly efficient in yeast. Finally, what makes yeast especially attractive is an extensive set of high-throughput tools that lend themselves to a systematic and genome-wide analysis of particular cellular processes or screenable phenotypes. These include not only comprehensive collections of yeast mutants with gene deletions [14], hypomorphic alleles, and conditionally repressible promoters [15], but also plasmid libraries that allow the study of gene overexpression [16] or systematic localization studies using the yeast GFP-fusion collection [17]. Using these high-throughput techniques in combination with recombinant expression of components implicated in the development of PD, the yeast model can be used to identify genes that positively and negatively regulate important processes linked to PD pathology, like protein aggregation and cellular toxicity.

## 3. Yeast Models for *α*-Synuclein and Synphilin-1

**Figure 1:**
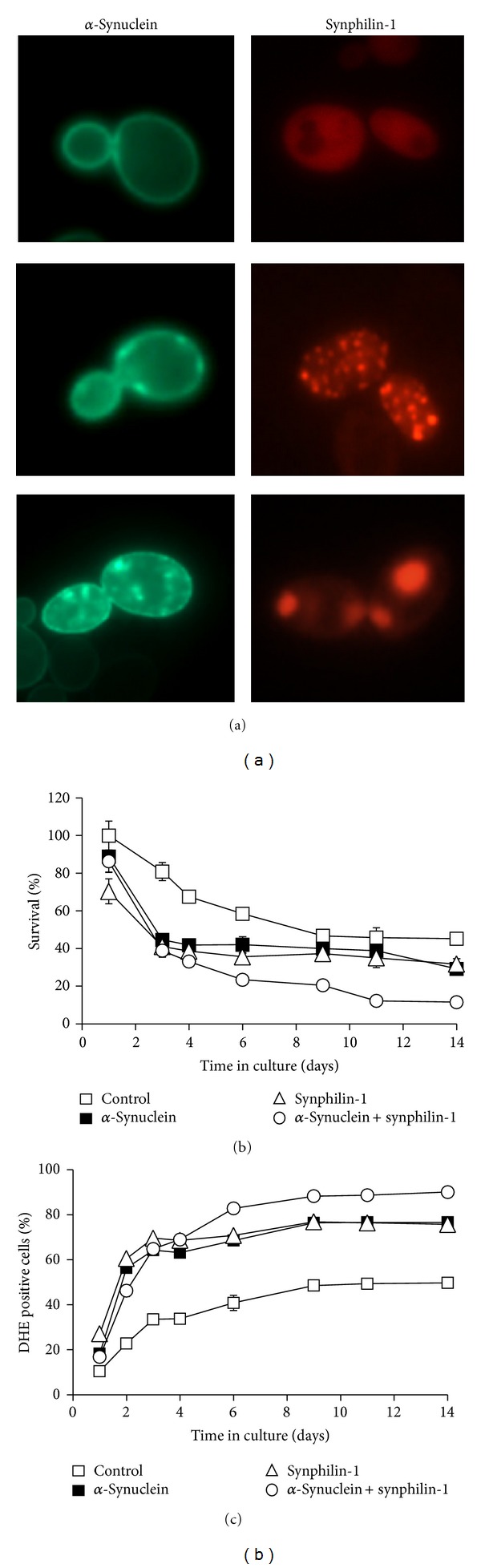
Expression of *α*-synuclein and synphilin-1 in *Saccharomyces cerevisiae*. (a) Fluorescence microscopic visualization of wild type *α*-synuclein-eGFP (left panels) and dsRed-synphilin-1 (right panels) fusion proteins expressed separately in wild type yeast cells. (b) and (c) Quantification of viable cells (b) and DHE positive cells (c) in wild type yeast cells expressing wild type *α*-synuclein and synphilin-1 alone or together. The strains were kept in culture for two weeks [24].

It has already been a decade since yeast was used for the first time as a model to study *α*-synuclein toxicity [18]. In this study, the intracellular localization of wild type and mutant *α*-synuclein fused to green fluorescent protein was visualized in yeast cells. At low expression levels, the wild type and A53T mutant fusion proteins accumulate at the plasma membrane, consistent with the affinity of *α*-synuclein for phospholipids [19]. However, upon increased expression, their localization shifted from the plasma membrane towards cytoplasmic aggregates (Figure 1(a), left panel). This coincided with an increase in toxicity reflected in a reduced growth of the yeast cells expressing these human proteins. A30P mutant *α*-synuclein on the other hand displayed a cytosolic localization, and a minor toxic effect could only be seen upon multicopy expression. Since the description of this initial model, several other groups have taken advantage of the potential that the yeast model system offers to study PD-related features. Beside studies concentrating on a specific cell biological process, also genome-wide screens have been conducted to identify genes and processes that modulate *α*-synuclein-induced toxicity in yeast. These studies demonstrate that *α*-synuclein interferes with a broad range of processes to exert its toxicity, like membrane binding, protein quality control and autophagy, Ca^2+^ and Mn^2+^ transport, protein phosphorylation, vesicular trafficking and recycling, and cell death and aging [20–23]. 

The protein-protein interaction between *α*-synuclein and synphilin-1 has originally been described using the yeast-two-hybrid system. Hence, the yeast model can probably also be used to study the functional interactions between these two proteins. In a humanized yeast model, a dsRed-synphilin-1 formed aggregates in about 30% of the cell population (Figure 1(a), right panel) [24]. This is much more than an *α*-synuclein-eGFP fusion, which induces aggregate formation in only 2% of the cells. However, when coexpressed, synphilin-1 induced a sixfold increase in the amount of cells with *α*-synuclein aggregates. When fluorescently tagged synphilin-1 and *α*-synuclein formed inclusions in the same cell, they would often colocalize, still, aggregates containing only one of the two proteins could also be found. The pathways leading to synphilin-1 or *α*-synuclein aggregates however seem quite different. While soluble synphilin-1 is localized mainly in the cytoplasm, its aggregates would form at lipid droplets and detergent resistant membranes (DRMs). In contrast, *α*-synuclein localizes mainly at the plasma membrane, where its inclusions also initiate [18, 25], but it interacts less with DRMs as compared to synphilin-1 [24]. 

### 3.1. The Reciprocal Relation between *α*-Synuclein and the Ubiquitin-Proteasome System

Healthy cells rely on quality control mechanisms that monitor the proper folding of native proteins and respond to damaged and misfolded proteins. These include molecular chaperones that stabilize and refold abberrant and damaged proteins and proteolytic systems that eventually remove proteins beyond repair. Despite the description of a decrease in proteasomal function in the substantia nigra in PD [26] and an accumulation of ubiquitin in Lewy bodies [27, 28], the precise role for the ubiquitin-proteasome system (UPS) (dys)function in the development of PD remains to be elucidated. For this reason the relationship between the ubiquitin-proteasome system and *α*-synuclein was also studied in more detail in the yeast models for PD. In their pioneering PD yeast model, Outeiro and Lindquist [18] reported about the link between *α*-synuclein toxicity and the ubiquitin-proteasome system by showing an increase in ubiquitin accumulation and decreased proteasomal function in yeast cells expressing *α*-synuclein. This *α*-synuclein-mediated proteasome impairment in yeast is not caused by changes in the individual peptidase activities of the proteasome or by the amount of available proteasome complexes [29]. Instead, impairment of the proteasome coincided with an altered proteasome composition. Next to the effect of *α*-synuclein expression on proteasomal function, also the inverse relation has been studied. The effect of proteasome activity on *α*-synuclein behaviour was investigated using the proteasomal inhibitor, lactacystin, or by the analysis of yeast mutants deleted for proteasomal components. These approaches demonstrated that malfunctioning of the proteasome increases the accumulation of *α*-synuclein inclusions [25, 30] and enhances *α*-synuclein toxicity [30, 31], indicating a role for the proteasome in *α*-synuclein degradation. Such proteasomal degradation of *α*-synuclein most likely occurs on the soluble form, since clearance of *α*-synuclein aggregates by the yeast proteasome is negligible [32]. 

### 3.2. Autophagic Clearance of *α*-Synuclein Aggregates

When the activity of the proteasome becomes compromised or overwhelmed, misfolded proteins are directed to the autophagic pathway for degradation. This pathway is well conserved among eukaryotic cells and involves the lysosome in mammalian cells and the vacuole in yeast. The observation in higher eukaryotes that *α*-synuclein aggregates can be removed by autophagy suggested a connection between autophagy and the pathogenesis of PD [33–35]. 

Autophagy induction in both mammals and yeast has previously been achieved by treatment with rapamycin, leading to the inhibition of the TOR kinase. Treating *α*-synuclein expressing yeast cells with this autophagy-inducing drug resulted in a significant decrease of *α*-synuclein inclusions [25]. In a recent study, yeast cells expressed multiple copies of *α*-synuclein-GFP under an inducible *GAL1*-promotor, allowing the study of the formation of *α*-synuclein aggregates on galactose-containing medium subsequent repression of *α*-synuclein expression, and a chase of the aggregates on glucose-containing medium [32]. This clearly showed that, once neosynthesis of *α*-synuclein is switched off, yeast cells can rid themselves of the formed aggregates within hours, which also rescues the *α*-synuclein cellular toxicity. This aggregate clearance seems to be mainly mediated by autophagy, as evidenced by pharmacologic inhibition with PMSF, which blocks vacuolar serine proteases, or via genetic blockade in a yeast mutant for Atg1, a kinase essential for the induction of autophagy. Furthermore, yeast Ypk9, the ortholog of a lysosomal P-type ATPase (ATP13A2) was identified as a suppressor of *α*-synuclein toxicity, and overexpression of Ypk9 was shown to reduce intracellular *α*-synuclein inclusions [22, 36]. In contrast with these studies reporting on clearance of *α*-synuclein inclusions via the autophagic-lysosomal pathway, it was observed that fluorescently tagged wild type and A53T mutant *α*-synuclein are not able to enter the vacuolar lumen in yeast and that expression of the native proteins causes a defect in vacuolar fusion [23]. The A30P mutant, on the other hand, did not affect vacuolar fusion and was targeted to the vacuole via the endocytic pathway upon overexpression of the *α*-synuclein toxicity suppressor Ypp1 [37].

### 3.3. Role of Mitochondrial Dysfunction and Mitophagy in *α*-Synuclein Toxicity

Mitochondria are not only the main producers of cellular energy but are also considered to be important regulators of neuronal functioning. Small irregularities in their normal function have been implicated in the pathogenesis of various neurodegenerative diseases, including PD. Early evidence for this came from observations that administration of mitochondrial complex I inhibitors, such as MPTP, rotenone, and paraquat, resulted in changes reminiscent of those seen in PD [38–40]. Later studies showed that several of the familial PD-associated proteins are linked directly or indirectly to mitochondrial pathways including PINK1, Parkin, DJ-1 and Omi/HtrA2 as reviewed in [41]. In addition, increasing evidence suggests that mitochondrial function is also affected by *α*-synuclein via multiple mechanisms. Interestingly, studies in yeast models identified genes and compounds affecting mitochondrial function as being critical mediators of *α*-synuclein toxicity [23, 42, 43].

Zabrocki and colleagues [23] performed a genetic screening by expressing an *α*-synuclein-eGFP-fusion protein in a genome-wide collection of viable yeast deletion strains. This screening retrieved 15 mutants that were deleted for a gene involved in mitochondrial function or the oxidative stress response. These included genes responsible for the induction of the antioxidant response or involved in the actual removal of the oxidating agents and a series of genes mediating the degradation of misfolded mitochondrial proteins. 

Subsequently, the importance of mitochondria in *α*-synuclein toxicity was also demonstrated in postmitotic, stationary phase yeast cells, via measurement of the so-called chronological life span (CLS) [44]. Expression of wild type and A53T mutant *α*-synuclein in these aged yeast cells, which rely on mitochondria for their energy production, induces a strong increase in markers for reactive oxygen species (ROS), apoptosis, and necrosis, eventually resulting in cell death. This effect is not dependent on the main apoptotic players or the unfolded protein response. Instead, in cells lacking functional mitochondria (rho^0^), *α*-synuclein was not able to exert this toxic effect and failed to induce cell death. In a study by the group of Lindquist, a microarray analysis was performed using a yeast model with *α*-synuclein overexpression [43]. This revealed a decrease in transcript levels of genes involved in mitochondrial and respiratory functions and an upregulation of transcripts related to oxidoreductase activities. Phenotypically, this manifested itself in an abnormal mitochondrial morphology, which correlated with a loss of mitochondrial membrane potential and a striking increase in ROS production. As such, these data confirm that *α*-synuclein overexpression strongly affects mitochondrial functions. 

Increasing insight in the physiological function of the familial risk genes PINK1 and Parkin has highlighted the importance of mitochondrial dynamics, more specifically their role in the regulation of the removal of damaged mitochondria via mitophagy (reviewed in Imai and Lu [45]). A recent study used the yeast PD model to examine the link between *α*-synuclein toxicity and mitophagy in chronologically aged yeast cells [46]. Expression of wild type and A53T mutant *α*-synuclein in yeast was shown to induce mitophagy. Moreover, the *α*-synuclein-mediated decrease in chronological life span is even dependent on mitophagy, since deletion of Atg32, a mitochondrial protein required for initiation of mitophagy in yeast, alleviated *α*-synuclein induced toxicity. This also correlated with lower ROS levels in these *atg32* deletion mutants upon *α*-synuclein expression.

Furthermore, the important role of mitochondria and ROS was also demonstrated in small-molecule screens using humanized yeast as a screening tool. A yeast strain overexpressing *α*-synuclein was used to screen a chemical library for compounds that are able to relieve toxicity [43]. Four 1,2,3,4-tetrahydroquinolines could reduce the *α*-synuclein toxicity at multiple levels, that is, by a reduction of the formation of *α*-synuclein aggregates, restoration of vesicular trafficking from ER to Golgi, and rescue of mitochondrial abnormalities. Earlier, a similar screen for compounds alleviating *α*-synuclein toxicity in yeast cells has been performed by Griffioen et al. [42]. From a library of about 10.000 small molecules, two could rescue the growth defect caused by *α*-synuclein overexpression, that is, epigallocatechin-3-gallate (EGCG) and quercetin. These two compounds are flavonoids with antioxidant properties, which are found in large amounts in green tea. 

### 3.4. *α*-Synuclein and Synphilin-1 Toxicity Is Sirtuin Dependent

Sirtuins or Silence Information Regulators (SIRs) have originally been discovered in yeast. They possess NAD-dependent protein deacetylase activity, which plays a key role in transcriptional silencing at genomic loci including the mating-type locus, telomeres, and ribosomal DNA (rDNA) [47, 48]. In yeast, Sir2 was shown to be required for the increased mitotic life-span, that is, the number of times a cell can divide, upon calorie restriction [49]. This is in contrast with its role in nondividing cells, where deletion of *SIR2* extends chronological life span (CLS), that is, the cell viability of a batch culture over a period of several weeks. Being evolutionary conserved from bacteria to humans, sirtuins were also found to modulate aging in multicellular organisms such as nematodes, fruit flies, and mice [50–52]. The association of sirtuins with aging made these proteins of great interest for researchers because of their potential as therapeutic targets for age-related diseases such as Parkinson's disease. 

The CLS model is typically used to study aging in yeast. In such cultures, expression of *α*-synuclein and synphilin-1 both induced a strong increase in ROS accumulation and a concomitant decrease in cell survival (Figures 1(b) and 1(c)) [24]. However, in a *sir2* deletion mutant, the toxicity induced by *α*-synuclein expression was lowered while for synphilin-1 it was largely absent. This suggests that these disease-associated proteins exert their effect via a Sir2-dependent process, possibly involving the segregation of these proteins. 

More recently, it was reported that Sir2 is an essential mediator of *α*-synuclein toxicity in aged yeast cells via its control of mitophagy [46]. The process of mitophagy, which is required for wild type and A53T mutant *α*-synuclein to execute its toxic effect, is blocked in a *sir2* deletion mutant.

### 3.5. Phosphorylation of *α*-Synuclein

Most of the *α*-synuclein found in Lewy bodies of PD patients is phosphorylated at Ser129 [53]. A large amount of research has gone into the elucidation of the role of Ser129 phosphorylation in the processes of *α*-synuclein localization, aggregation, and toxicity. Still, there is no clear consensus since studies in animal models of PD have yielded conflicting results on the role of Ser129 phosphorylation [54–56]. It has been shown that Ser129 can be phosphorylated by several kinases, like the polo-like kinases (PLK) 1 to 3, casein kinases (CK) 1 and 2, and the leucine-rich repeat kinase 2 (LRRK2) [57–59]. The polo-like kinases and casein kinases possess yeast orthologues, allowing their study in this simple model system. Zabrocki and coworkers [23] expressed *α*-synuclein in yeast mutants deleted for the yeast casein kinases. Deletion of the plasma membrane localized CK-1 kinases, Yck1 or Yck2, resulted in decreased Ser129 phosphorylation and reduced toxicity in yeast cells. Deletion of Yck3, a CK-1 kinase localized at the vacuolar membrane influenced neither the *α*-synuclein phosphorylation level nor its toxicity. However, Yck3 was picked up as a component that, when overexpressed, reduced the *α*-synuclein toxicity in yeast, indicating that the influence of *α*-synuclein phosphorylation on toxicity might depend on the subcellular localization of the kinase and the *α*-synuclein substrate [22]. It should be noted however that biochemical evidence that these yeast CK-1 kinases directly phosphorylate *α*-synuclein is still lacking. On the other hand, a recent study also showed that deletion of Yck1 or Yck2 increased the *α*-synuclein toxicity, while, similar to Yck3, overexpression of Yck1 could partially alleviate the *α*-synuclein induced growth defect [60]. 

Overexpression of Cdc5, the yeast polo-like kinase 2, also rescues *α*-synuclein toxicity [22]. However, a detailed analysis of the relationship between the Cdc5 kinase and *α*-synuclein toxicity in yeast gave quite surprising results [61]. For this kinase, good biochemical evidence was provided that it directly phosphorylates the Ser129 residue. However, *α*-synuclein was shown to be toxic to yeast cells but not because it was phosphorylated at this residue. Instead, *α*-synuclein inhibits the Cdc5 kinase from binding and activating Tus1, a guanine nucleotide exchange factor for the small GTPase Rho1, and thus disrupts the signaling cascade it controls, the so-called cell wall integrity pathway. This MAP kinase pathway is not only regulated through the cell cycle but is also responsive to a number of external stressors that act upon the cell wall [62]. These results could be confirmed in mammalian cells that, unlike yeast cells, do not have cell walls but do contain similar PLK-Rho/Ras-MAPK signaling modules that are activated upon cell stress. In a neuroblastoma cell line, *α*-synuclein expression was shown to cause an attenuated stress-induced activation of the p38 and JNK MAP kinases, resulting in reduced cell viability [61]. These effects of *α*-synuclein toxicity and impaired MAPK signaling were abolished in an *α*-synuclein mutant that cannot bind the mammalian polo-like kinase 2. 

### 3.6. Increased Intracellular Ca^**2+**^ Mediates *α*-Synuclein Toxicity

Ca^2+^ is an important intracellular messenger that regulates a variety of vital cell functions. Therefore, its levels are tightly controlled in the cytoplasm, ER, and mitochondria. Accumulating evidence points towards an important role for Ca^2+^ imbalance in the pathogenesis of neurodegenerative diseases such as PD [63, 64]. Although several of these studies linked Ca^2+^to *α*-synuclein toxicity and aggregation, the underlying mechanisms remain unclear [65–67]. Given the high conservation of the regulation of Ca^2+^ homeostasis between yeast and humans, yeast models were recently used to investigate this matter [68]. Heterologous expression of *α*-synuclein in yeast elicited an increase in cytosolic calcium levels, which preceded a rise in oxidative radicals and eventually cell death. Systematic deletion of the calcium channels, buffering proteins, and sensors known from the yeast genome pointed to a role for the Golgi-resident Ca^2+^/Mn^2+^ ATPase *PMR1* (plasma membrane-related Ca^2+^ATPase1). Its deletion decreased the *α*-synuclein-induced elevation in cytoplasmic Ca^2+^ levels and also led to a strong decrease in ROS production and subsequent cell death. Furthermore, in the study of Büttner et al. [68], the *PMR1* orthologues in flies and nematodes were also shown to be required for an *α*-synuclein induced Ca^2+^ increase, leading to loss of dopaminergic neurons. A similar intracellular Ca^2+^ buildup has recently been described in a transgenic mouse model, where overexpression of *α*-synuclein seems to interfere with cytosolic calcium clearance and buffering mechanisms [69]. Increased Ca^2+^ levels, leading to mitochondrial oxidative stress, could provide an explanation for the preferential loss of dopaminergic neurons of the substantial nigra pars compacta in PD. Work by the group of Surmeier has shown that, because of their specific dependence on L-type Ca^2+^ channels for autonomous pacemaking, higher intracellular Ca^2+^ levels render these cells specifically vulnerable to oxidative damage [70, 71].

### 3.7. Segregation of *α*-Synuclein and Synphilin-1 Aggregates

Although aggregation of disease-associated misfolded proteins such as *α*-synuclein and synphilin-1 has already been scrutinized in a large number of studies, many fundamental questions remain. Recently, the use of yeast to study the role of the cytoskeleton as a transport route directed towards large protein aggregates has become an important area of focus. This research revealed that protein aggregates are not random deposits of insoluble material but are formed via an active and regulated process, involving transport of small deposits along components of the cytoskeleton, that is, microtubuli and actin cables. In this respect, it has been observed that the so-called aggresomes are formed by the convergence of small inclusions at the centrosome via microtubuli-based transport [72]. These aggresomes are thought to be cytoprotective since they can easily be removed via autophagy. Other studies provided data suggesting that during yeast cell division, damaged and aggregated proteins are asymmetrically segregated between mother and daughter cells in a Sir2, polarisome, and actin-cytoskeleton-dependent manner [73, 74]. 

When expressed in yeast, *α*-synuclein has been shown to be initially localized at the plasma membrane [18, 25]. At this site, the protein starts to form small aggregates, which later evolve to larger cytoplasmatic inclusions. Synphilin-1, on the other hand, starts out being dispersed in the yeast cytoplasm and forms a number of small cytoplasmatic aggregates, which evolve into one or a few large cytoplasmic inclusions [24]. Interestingly, screening the genome-wide collection of yeast deletion strains to identify mutants that display enhanced inclusion formation of *α* synuclein eGFP, retrieved 24 mutants that are affected in genes involved in tubulin, actin, and cytoskeleton functions. These include the major components of the polarisome (Bni1, Pea2, Spa2, and Sph1), which is a focal point of actin polymerisation during yeast cell division as well as the GimC/prefolding complex, which is required for efficient transfer and folding of newly synthesized actin and tubulin by the chaperonin TriC/CCT [23]. The outcome of this screen suggests that the transport of *α*-synuclein aggregates might also be dependent on the actin/polarity machinery [75]. Analysis of the synphilin-1 inclusions in yeast demonstrated that the large inclusions, which were observed in stationary phase cells, correspond to aggresomes [24]. In addition, it was found that synphilin-1 inclusions localized to actin cables and actin patches. Moreover, selective drug-induced disruption of the structure of actin filaments and microtubuli by addition of, respectively, Latrunculin-B and Benomyl, revealed that the transport of synphilin-1 inclusions along actin cables is equally important to prevent synphilin-1 toxicity as aggresome formation via microtubuli-mediated transport. Furthermore, the observation that Sir2 is required for synphilin-1 to exert its toxic effect together with its role to retain damaged and aggregated proteins in the yeast mother cell suggests that Sir2 might also be important for the segregation of synphilin-1 aggregates in the described yeast PD model. Together, these findings strengthen the role of the cytoskeleton and Sir2 in the transport of synphilin-1 aggregates and point towards a possible role of the elements of the cytoskeleton in the segregation of *α*-synuclein aggregates. 

## 4. Concluding Remarks

Over the past ten years, several research groups have developed great expertise in uncovering the cellular aspects of *α*-synuclein toxicity using humanized yeast models. More recently, a yeast model was also designed to study the presumed pathobiology of the *α*-synuclein interaction partner synphilin-1. 

Despite its limitations as a unicellular eukaryote, yeast can faithfully reproduce key features of PD pathology. Moving on from studying mere protein aggregation and growth inhibition, these models now start to provide a tool to study new features of the *α*-synuclein induced-cellular toxicity. One of the new advances that has been studied in yeast addresses the role of an intracellular Ca^2+^ buildup upon *α*-synuclein expression, mediated by the plasma membrane-related Ca^2+^ATPase1. This Pmr1-induced Ca^2+^ increase appears to be essential for *α*-synuclein toxicity from yeast to flies and nematodes. Furthermore, the yeast polo-like kinase 2, Cdc5, which was thought to induce *α*-synuclein toxicity by phosphorylating Ser129, appears to be inhibited itself by *α*-synuclein, leading to reduced cell wall integrity signaling. Similarly, an *α*-synuclein-induced reduction of PLK2 signaling, resulting in inhibition of MAPK signaling, was also shown to increase stress sensitivity in mammalian cells. Finally, yeast has given some clues that *α*-synuclein-induced toxicity is dependent on the process of mitophagy, which has recently also been implicated in the PD pathology mediated by human PINK and Parkin-1. These results demonstrate the usefulness of humanized yeast models in uncovering new molecular and cellular attributes of *α*-synuclein and synphilin-1 toxicity.
